# Pediatric Nephrolithiasis

**DOI:** 10.3390/healthcare11040552

**Published:** 2023-02-13

**Authors:** Brent Cao, Roby Daniel, Ryan McGregor, Gregory E. Tasian

**Affiliations:** 1 Department of Urology, College of Medicine, The University of Illinois, Chicago, IL 60612, USA; 2 Department of Medicine, The Perelman School of Medicine at the University of Pennsylvania, Philadelphia, PA 19104, USA; 3 Division of Urology, The Children’s Hospital of Philadelphia, Philadelphia, PA 19104, USA

**Keywords:** nephrolithiasis, pediatrics, kidney stone disease, ureteroscopy, percutaneous nephrolithotomy, shock wave lithotripsy, radiation exposure, epidemiology, ultrasound

## Abstract

The prevalence of pediatric nephrolithiasis has increased dramatically in the past two decades for reasons that have yet to be fully elucidated. Workup of pediatric kidney stones should include metabolic assessment to identify and address any risk factors predisposing patients to recurrent stone formation, and treatment should aim to facilitate stone clearance while minimizing complications, radiation and anesthetic exposure, and other risks. Treatment methods include observation and supportive therapy, medical expulsive therapy, and surgical intervention, with choice of treatment method determined by clinicians’ assessments of stone size, location, anatomic factors, comorbidities, other risk factors, and preferences and goals of patients and their families. Much of the current research into nephrolithiasis is restricted to adult populations, and more data are needed to better understand many aspects of the epidemiology and treatment of pediatric kidney stones.

## 1. Introduction

Urolithiasis is a condition caused by the formation or presence of mineral deposits (also known as calculi or stones) in the urinary tract. Urolithiasis can be further divided into nephrolithiasis (deposits in the kidney or kidney stone), ureterolithiasis (ureteral stone), and cystolithiasis (bladder stone). When urolithiasis occurs in a child (an individual less than 18 years of age), this is known as pediatric urolithiasis [1].

## 2. Evolving Epidemiology

**Figure 1:**
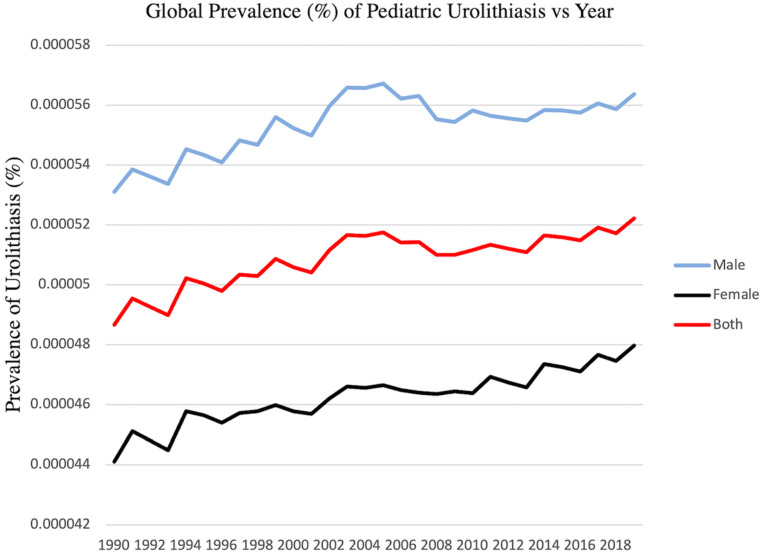
Line graph showing the global prevalence (%) of pediatric urolithiasis from 1990 to 2019. Data from the Global Burden of Disease Collaborative Network [11].

Nephrolithiasis affects approximately 11% of the United States population, a number that continues to increase along with associated healthcare expenditures [2,3]. However, pediatric kidney stone disease is less common with an estimated prevalence of about 1% [4]. Population-based studies in the United States estimate the incidence of pediatric urolithiasis to be around 65/100,000 person years during the 2005–2016 period, a sharp increase from the 1999 estimate of 18/100,000 person years [5,6]. Similarly, the global prevalence of urolithiasis is increasing, perhaps due to changes in diet, climate, comorbidities, and living conditions [7,8,9,10]. Figure 1 shows the global prevalence (%) of pediatric urolithiasis from 1990 to 2019, stratified by sex, based on data from the Global Burden of Disease Collaborative Network (Figure 1) [11]. These findings are in line with observations that modern pediatric stone disease is more likely due to a change in the exposome rather than underlying genetic or systemic diseases [12].

### 2.1. Differences by Sex

By the second decade of life, girls have a higher frequency of stones compared to boys [3,13]. Similarly, adolescent females had a 28% increase in incidence rates of nephrolithiasis per 5 years, the greatest increase of nephrolithiasis across all age groups and sex [3]. Prior reviews exploring this trend discussed how boys were more likely to have metabolic disorders, were more susceptible to warm weather, and how estrogen may provide a protective environment against nephrolithiasis, which all contrasts the increasing kidney stone incidence in adolescent girls [14]. Some studies reviewed stone phenotypes but did not find significant differences. Nonetheless, a likely unknown risk factor is responsible for the excess of female pediatric urinary stones [13].

### 2.2. Differences by Race

With regards to race, pediatric nephrolithiasis appears to mirror trends seen in adults with stone disease being most common among non-Hispanic Caucasians followed by Hispanic Caucasians and African-Americans [5,15,16]. However, African-Americans showed the largest increase in incidence based on a 20-year population study in South Carolina [3]. Nevertheless, the epidemiology of pediatric kidney stone disease has not been as rigorously studied as that of adult stone disease, especially with regards to race.

### 2.3. Risk of Recurrence

With more years at risk for stone recurrence, children with kidney stones comprise a high-risk patient population requiring focused attention [17]. Some studies estimate the probability of symptomatic stone recurrence to be 50% within 3 years of the stone event even with intervention, a rate higher than that reported for adults [18]. Without any intervention, the 3-year recurrence rate was as high as 75% [19]. Studies also explored various preventative strategies with conflicting results. However, the general consensus appears to be that preventative management should be individualized to each patient, often with a focus on supportive and/or lifestyle changes such as a Dietary Approach to Stop Hypertension eating plan (DASH), drinking plenty of fluids, and limiting fructose intake [18,20,21,22,23]. Medical management with thiazide diuretics or potassium citrate may also be indicated, and completing a 24-h urine collection has been associated with a significant (60%) reduction in stone recurrence [18]. Newer studies also suggest that the stone recurrence rate is increasing over time, which may be due to warming weather, antibiotic exposure, supplement use, or other unidentified factors [24].

### 2.4. Stone Composition

Compared with stones formed during adulthood, current studies suggest that childhood stone distribution is similar with 70–80% being calcium oxalate, 10–15% being struvite, 10% being calcium phosphate, and <5% being uric acid [17]. These findings reflect increased calcium phosphate stones and decreased uric acid stones, which may be due to the more alkaline urine that children produce [25]. While many children do indeed have inborn errors of metabolism or anatomic abnormalities that contribute to stone disease, most cases are likely related to metabolic risks such as hypercalciuria [12].

### 2.5. Extrarenal Manifestations

Patients with nephrolithiasis are at risk for the development of comorbidities such as decreased bone mineral density, chronic kidney disease, and cardiovascular disease [26]. Low bone mineral density and defects in bone remodeling have been commonly observed with nephrolithiasis in general. Similarly, children with stone disease are at a higher risk of having decreased bone mineral density and fractures [27,28]. A population-based cohort study of patients in the United Kingdom found that among those with nephrolithiasis, the highest risk of fracture was observed among boys aged 10–19 years old. While a causal mechanism has not been identified, some suggest that hypercalciuria and a negative calcium balance may be related to low bone mineral density and increased fracture risk [29]. Since childhood is a crucial period for bone growth, special attention should be paid to bone mineral density to prevent complications later in life [30,31].

A Canadian cohort study of over 1.9 million patients found that stone episodes were associated with an over 200% increased risk of end-stage renal disease (ESRD) [32]. Another cohort study found a 170% increased risk of incident chronic kidney disease for patients with urolithiasis [33]. Although these studies were conducted in adult populations, children may be particularly susceptible due to early onset of disease—more years at risk for chronic kidney disease and recurrent stone formation—that reflects a more severe phenotype. An early study conducted in a tertiary care center in Turkey found that multiple stone formers were over eight times more likely to develop chronic renal failure [34].

Hypertension was also found to be associated with nephrolithiasis, likely due to a mechanism beyond the increased risk from a high-sodium diet [35]. Additionally, other mechanisms such as alterations in renal calcium and sodium handling can contribute to both nephrolithiasis and hypertension. Finally, shockwave lithotripsy may also be associated with hypertension, which complicates identifying whether hypertension is related to nephrolithiasis, iatrogenic treatments such as shockwave lithotripsy, or both [26].

### 2.6. Burden of Disease

The burden of pediatric nephrolithiasis mirrors the changing epidemiology. In the United States, the hospital costs for inpatient care increased 20% from 1997 to 2012 [13]. In 2015, the net economic burden of American hospital care for childhood stone formers was estimated at USD 375 million per year (USD 229 million for admission and USD 146 million for emergency department charges) [36]. Over the same time period, pediatric nephrolithiasis discharges increased by 18%, and emergency department visits increased by 9% [37]. The true economic burden is likely higher when considering outpatient management, medications, and diagnostic workup such as medical imaging, especially since the vast majority of patients are managed non-operatively (approximately 85%). A 2022 analysis estimated that outpatient management accounted for about 67% of spending, while inpatient services accounted for about 52% [38].

The health care burden remains especially heavy in low-resource settings across the globe. Hot climates, poor nutrition, and other comorbidities such as gastrointestinal diseases are factors that contribute to the high burden in many countries such as Nepal, Tunisia, and Pakistan. In many cases, failure to obtain appropriate healthcare results in further economic burden due to complications such as renal failure, sepsis, and open surgery. Considering that children represent a patient group with a high likelihood of recurrence and more years for recurrence, the subsequent healthcare expenditures incurred by this patient group may be additionally understated [39].

## 3. Diagnostic Evaluation

### 3.1. Medical History and Physical Examination

As is imperative for all patients, a thorough medical history and physical examination are important in the evaluation of nephrolithiasis in children.

In order to determine the risk status of calculus formation amongst children, it is important to acquire an extensive dietary history. This includes information on (1) fluid intake (as lower urine volume effectively increases solute concentration which in turn increases the risk of nucleation), (2) overall sodium content (as a high-sodium diet will decrease RAAS activation which results in lower sodium absorption and increased excretion of calcium in the urine), (3) vitamin and mineral supplementation (as increased vitamin C consumption and its subsequent metabolism would increase oxalate levels), and (4) special diets (as increasing protein consumption in the ketogenic or paleo diet results in increased acid loads which downregulates calcium channels in the distal nephron responsible for calcium reabsorption) [40,41].

A medication list should be obtained in order to rule out the use of certain drugs that may induce the formation of metabolic calculi such as loop diuretics (furosemide), carbonic anhydrase inhibitors (acetazolamide), anti-epileptics (topiramate, zonisamide), and, when abused, laxatives [42]. In addition, medications can cause the formation of stones due to the supersaturation of the drug in urine such as magnesium trisilicate, ciprofloxacin, sulfa medications, triamterene, indinavir, and guaifenesin/ephedrine [42]. Other important history findings include prematurity, recurrent urinary tract infections, intestinal malabsorption, causes of neurogenic bladder such as spina bifida and spinal cord injury, known renal or ureteral anomalies, prior urinary tract reconstruction, and prolonged periods of remaining non-ambulatory [43,44].

A detailed family history of nephrolithiasis can be useful in the identification of inherited metabolic or genetic conditions that lead to stone formation such as cystinuria and primary hyperoxaluria [45].

Finally, physical examination looking for dysmorphic features, rickets, and tetany can provide clues to a hypercalciuric state caused by a metabolic or genetic condition. In addition, the presence of gout would point toward hyperuricemia as a result of enzyme dysfunction [46,47].

Although some children may present asymptomatically, abdominal or flank pain is usually the chief complaint upon evaluation, especially in the case of obstructing ureteral stones [48,49]. Other signs and symptoms on clinical presentation that should increase suspicion of nephrolithiasis include gross hematuria, dysuria, urgency, nausea, and vomiting [50].

### 3.2. Imaging

According to the European Association of Urology (2022 EAU Guidelines on Urolithiasis), ultrasonography (US) is the ideal primary imaging method that should be used when suspicious of nephrolithiasis in the pediatric population. US imaging should evaluate the kidney, fluid-filled bladder, and ureter. If diagnostic information cannot be attained with ultrasound, a kidney–ureter–bladder radiography (KUB) or a low-dose non-contrast-enhanced computed tomography (NCCT) may be utilized to capture more information. This practice would be in line with the Recommendations of the International Commission on Radiological Protection and Image Gently Alliance’s campaign to reduce radiation dose in diagnostic imaging for children [51,52,53].

The European Society of Pediatric Urology (ESPR) Pediatric Uroradiology Taskforce and the European Society of Urogenital Radiology (ESUR) Pediatric Uroradiology Working Group defined US criteria for a diagnosis of urolithiasis amongst children as an echogenic or hyperechoic focus with the generation of acoustic shadows (note: in comparison to conventional sonography, spatial compounding and tissue harmonic imaging do not produce shadows for all stones, especially those calculi less than 4 mm) [54,55]. To improve accuracy in the detection of calculi, the twinkling artifact or the color comet-tail artifact on color Doppler US has been suggested as it demonstrates a rapid and intense alternation in color signal just deep to a stone [56,57,58]. In order to elicit improved visualization of this artifact, color scale on the US machine should be set to the highest levels with filter and pulse repetition frequency increased to their maximal levels as this ensures reduction or elimination of color signal from blood flow in neighboring vasculature [59]. In cases of acute unilateral obstruction by a calculus, duplex Doppler US can be beneficial in its diagnostic capability as it would demonstrate an ipsilateral elevation in the resistive index [56,60].

In a prospective study evaluating the precision of ultrasonography, US was reported to have 76% sensitivity with 100% specificity in the detection of urolithiasis in children [53]. Upon further analysis, the lower sensitivity rating was due to US not identifying clinically insignificant stones of smaller caliber in the kidney or distal ureter [53]. Sensitivity for ureteral stone detection may also be improved by combining US with kidney–ureter–bladder radiography (KUB) as this would allow for better stone localization [53,56].

In comparison to ultrasonography, low-dose non-contrast-enhanced computed tomography (NCCT) has nearly 99% sensitivity and 98% specificity. Despite the greater sensitivity for detecting calculi, the increase in cancer risk in children supports the utilization of CT imaging as the second-line and alternative method to US when diagnosis cannot be met with ultrasonography despite high clinical suspicion [61,62,63]. Yet, in a study reporting trends in imaging for pediatric urolithiasis from 1999 to 2008, CT use increased especially amongst pediatric patients of older age and nonwhite race [64]. Although a single CT scan does not attribute much risk of cancer, repeat studies would increase the risk with conceivably high aggregate effective doses [63]. Recent changes in CT protocol using ultra-low dose and low-dose techniques still yield high sensitivity and specificity but are less effective in the detection of stones under 3 mm in size or in patients of higher BMI [65].

### 3.3. Metabolic Investigation

In a 2002 retrospective review studying the clinical outcome of pediatric stone disease, 50% of the patient population under the age of 10 years presented with nephrolithiasis as a result of a metabolic disorder. These children were nearly five times as likely to present with recurrent stones when compared to their counterparts without a metabolic disorder [66]. Therefore, it is important that a metabolic evaluation is completed for children with suspected nephrolithiasis. This should begin with stone analysis, when possible, as the identification of either a cystine or struvite stone with X-ray diffraction, infrared spectroscopy, or polarization microscopy would be diagnostic [67]. In addition, serum levels of calcium, phosphorus, bicarbonate, magnesium, and uric acid should be collected, along with urinary data of volume, pH, sodium, creatinine, citrate, calcium, oxalate, uric acid, and cystine. Although 24-h urine collection would be preferred, spot urine samples would also be appropriate [68]. Table 1 summarizes normal values of analytes taken for a 24-h urine sample across various age groups (Table 1) [69].

#### 3.3.1. Hypercalciuria

Amongst pediatric patients with nephrolithiasis, hypercalciuria rates were found to be between 33.8 and 42% [70,71]. For children of all age groups, urinary calcium levels of greater than 4 mg/kg in a 24-h urine sample would be defined as hypercalciuria. Spot urine sample values for calcium should be adjusted with patient age as the calcium/creatinine ratio decreases over the years [67].

Hypercalciuria has many different etiologies, but the most common cause amongst the pediatric population is idiopathic hypercalciuria. In a 2014 study of pediatric patients with urinary-system-related symptoms, 47.7% of subjects had idiopathic hypercalciuria [72]. Idiopathic hypercalciuria is characterized by normocalcemia in the absence of other diseases that could cause hypercalciuria. Although precise genes that contribute to idiopathic hypercalciuria have not been identified, the condition seems to arise as an interplay between several genes and the environment, and it follows an autosomal dominant pattern of inheritance with incomplete penetrance [73,74,75]. In order to definitively diagnose a patient with idiopathic hypercalciuria, several other conditions that cause normocalcemic hypercalciuria should be excluded such as hereditary hypophosphatemic rickets with hypercalciuria [76].

#### 3.3.2. Hyperoxaluria

Hyperoxaluria is a condition of elevated oxalate levels in urinary excretion because of increased endogenous production of oxalate in the liver (primary hyperoxaluria) or increased intestinal absorption of oxalate or increased dietary intake of oxalate and its metabolic precursors (secondary or enteric hyperoxaluria) [77]. Normal urinary oxalate levels in a 24-h urine sample are below 45 mg/1.73 m^2^ for children of all age groups, except children below one year of age whose normal urinary oxalate levels are below 13 mg/1.73 m^2^. Spot urine sample values for oxalate should also be adjusted with patient age as the oxalate/creatinine ratio in children decreases with age from 260–288 mg/mg for children under the age of six months to 32 mg/mg for children over 16 years of age [67].

Increased endogenous synthesis of oxalate in the liver can be due to one of three rare autosomal recessive disorders of primary hyperoxaluria. Recurrent episodes of nephrolithiasis, progressive nephrocalcinosis, and end-stage renal failure due to oxalate deposition are common manifestations of severe hyperoxaluria (often defined as more than three times upper limit normal) [78]. Although there have been recent advancements in the management of patients with primary hyperoxaluria, diagnosis of this condition has been quite difficult due to the heterogeneity of the clinical presentation.

Of the primary hyperoxalurias (PHs), primary hyperoxaluria type 1 (PH1) is the most common form, responsible for nearly 80% of PH cases [77]. PH1 is due to AGXT gene mutations on chromosome 22 which results in a defect of the pyridoxine-dependent peroxisomal liver enzyme, alanine glyoxalate aminotransferase (AGT). This hepatic enzyme is responsible for the transamination of L-alanine and glyoxalate to pyruvate and glycine. When AGT activity is no longer present, glyoxylate is converted to oxalate via glycolate oxidase [79]. In November of 2020, Lumasiram (OxlumoTM), a small interfering RNA (siRNA) against the mRNA for the hydroxyacid oxidase 1 (HAO1) gene that encodes glycolate oxidase was approved for the treatment of all patients with PH1, including children [80]. Primary hyperoxaluria type 2 (PH2) accounts for about 10% of PH cases and is due to mutations in the GRHPR gene on chromosome 10 which results in enzymatic dysfunction of glyoxalate reductase-hydroxypyruvate reductase (GRHPR) [81,82]. Primary hyperoxaluria type 3 (PH3) is responsible for the remaining 10% of PH cases and is due to mutations in the HOGA1 gene on chromosome 9 which results in the dysfunction of mitochondrial 4-hydroxy-2-oxoglutarate aldolase [83]. Since PH1, PH2, and PH3 all present with elevated urinary oxalate levels, the increase in urinary concentrations of glycolate in PH1, glycerate in PH2, and 4-hydroxy-2-oxoglutarate in PH3 is a good method by which to differentiate each form of PH. If the urinary levels of these metabolites are normal, secondary hyperoxaluria should be considered.

Secondary hyperoxaluria is caused by an increase in intestinal absorption of oxalate (enteric oxaluria) or an excess in the dietary intake of metabolic precursors of oxalate or oxalate itself [77]. Enteric oxaluria can be caused by conditions that lead to fat malabsorption such as inflammatory bowel diseases, cystic fibrosis, or celiac disease [84]. Since calcium binds free fatty acids in the intestinal lumen, oxalate loses its counterion resulting in an increase in gut absorption of oxalate and excretion in the urine [85]. Thus, less calcium in the diet can also lead to increased oxalate absorption in enteric oxaluria. In addition, diets high in ascorbic acid, the precursor of oxalate, and oxalate are other causes of secondary hyperoxaluria.

#### 3.3.3. Hypocitraturia

Citrate is an important inhibitor of calculus formation in the urine as it (1) prevents the binding of calcium with other counter ions such as oxalate and phosphate via calcium–citrate complexation, (2) prevents the aggregation and growth of crystals by binding to the crystal’s surface, and (3) increases the pH of urine which increases the solubility of calcium oxalate [86,87,88]. Thus, hypocitraturia is a significant risk factor for nephrolithiasis. Hypocitraturia is defined as less than 365 mg/1.73 m^2^ in male children and less than 310 mg/1.73 m^2^ in female children in a 24-h urine sample [67,89,90]. Normal spot urine sample values for citrate/creatinine decrease from 0.42 g/g to 0.25 g/g after 5 years of age [91]. This decrease in urinary citrate is primarily due to intracellular acidosis of the nephron’s proximal tubule, which ultimately results in increased reabsorption of citrate. Although hypocitraturia is usually idiopathic by origin, conditions that cause metabolic acidosis and hypokalemia such as distal renal tubular acidosis should be considered. Moreover, high-protein, low-alkali diets that emphasize animal protein and neglect vegetable fiber and potassium can result in hypocitraturia [92,93].

#### 3.3.4. Cystinuria

Cystinuria is the most common cause of inherited nephrolithiasis and represents nearly 6% to 8% of all pediatric stones [94]. Cystinuria is an autosomal recessive genetic disorder caused by SLC3A1 gene mutations on chromosome 2 and/or SLC7A9 gene mutations on chromosome 19 [95]. This results in a defect in the reabsorption of cystine along with ornithine, lysine, and arginine at the proximal renal tubule. At lower pH levels, cystine is relatively insoluble causing its precipitation in the renal collecting system. Normal urinary cystine levels are below 13 mg/1.73 m^2^ for children under the age of 10 and below 48 mg/1.73 m^2^ for children over the age of 10 [67]. Fifty percent of patients with cystinuria present with their first stone by age 10, and another twenty-five percent of patients present in their teenage years [96,97]. If preventive measures are not taken, patients will suffer from recurrent stone episodes which, in the long term, can result in renal insufficiency and chronic kidney disease [94,97].

#### 3.3.5. Hyperuricosuria

In a recent study of pediatric patients with upper urinary tract calculus formation, only 5% of stones were purely uric acid [17,98]. The most significant risk factor for the crystallization and formation of uric acid stones is low urinary pH, especially below 5.5, as this results in decreased solubility of uric acid [99]. This lower urinary pH can be due to several causes including decreased ammonia production as seen in diabetes, decreased citrate intake, and systemic acidosis. For children between 1 and 5 years of age, normal urinary levels of urate in a 24-h urine sample should be below 11 mg/kg, while children over the age of 5 should have urate levels below 9.3 mg/kg. If spot urine sample values are to be used, the uric acid/creatinine ratio should be multiplied by plasma creatinine levels since normal values for children over the age of 2 are characterized as greater than 0.56 mg/dL per GFR [67].

Children with hyperuricosuria can present with recurrent stone episodes or even acute renal failure from urate crystal nephropathy because of inherited defects of enzymes associated with purine metabolism such as hypoxanthine-guanine phosphoribosyltransferase (HPRT), adenine phosphoribosyltransferase (APRT), xanthine dehydrogenase (XDH), or phosphoribosylpyrophosphate synthetase [100]. Renal failure with hyperuricemia can also present in children treated for leukemia and lymphoma [100]. In addition, hyperuricosuria is seen in hereditary renal hypouricemia due to SLC22A12 and SLC2A9 gene mutations which cause an impairment in the renal proximal tubular reabsorption of urate [101,102,103].

## 4. Medical Management

Management of pediatric nephrolithiasis includes management of acute stone episodes as well as prevention of disease recurrence. Prevention of disease recurrence can be accomplished with diet modification and pharmacological intervention, while acute management includes observation with supportive care, medical expulsive therapy (MET), and surgical intervention. The approach to acute management depends on several factors, such as stone location, size, likelihood of stone passage, severity of pain, and risk of infection or obstruction. The American Urological Association (AUA) guidelines for management of kidney stones recommend that pediatric patients presenting with asymptomatic and non-obstructing renal stones be offered evaluation for underlying metabolic abnormalities, assessment of urinary stone risk parameters, and periodic US surveillance with emphasis to parents or caregivers on the importance of regular follow-up [104]. Patients with uncomplicated ureteral stones ≤10 mm should be offered “observation with or without MET using α-blockers”. This recommendation is made based on evidence suggesting that MET is both effective in facilitating large stone passage in adult distal ureteral stones and safe in pediatric populations. In particular, a meta-analysis conducted by Hollingsworth et al. found that treatment of larger stones (defined variably as ≥ 5 mm, >5 mm, >6 mm, or >8 mm) with an alpha blocker in adult populations had a pooled 57% higher risk of stone passage compared with controls, and treatment of lower ureteral stones had a pooled 49% higher risk of stone passage compared with controls [105]. Furthermore, Ye et al. reported a reduction in time to stone expulsion, a decreased need for analgesic therapy, and relieved renal colic in adult patients, aged 18 to 60, who presented with distal ureteral stones and were treated with tamsulosin in a placebo-controlled, double-blinded study [106]. However, there is discordance in the literature regarding the efficacy of MET in facilitating stone passage. For example, the findings of a randomized clinical trial by Meltzer et al. did not support the use of tamsulosin in the treatment of urinary stones <9 mm [107], and a multicenter, randomized, placebo-controlled trial by Pickard et al. found that “tamsulosin 400 μg and nifedipine 30 mg are not effective at decreasing the need for further treatment to achieve stone clearance in 4 weeks for patients with expectantly managed ureteric colic” [108]. Further research is needed to determine the efficacy of alpha blockers in facilitating stone passage in pediatric populations, but the safety of MET in pediatric populations has been sufficiently proven to permit its use in the treatment of all ureteral stones [104]. Clinicians are advised to limit this trial of conservative therapy to a maximum of six weeks from clinical presentation in order to minimize the risk of irreversible kidney injury in the case of failure of stone passage [104].

## 5. Surgical Management

The AUA recommends surgical intervention in pediatric patients who present with symptomatic renal stones or with ureteral stones that are unlikely to pass spontaneously or have failed to pass after a trial of conservative therapy [104]. Just as in adult populations, there are three minimally invasive surgical methods available to pediatric patients: ureteroscopy (URS), shockwave lithotripsy (SWL), and percutaneous nephrolithotomy (PCNL). These methods come with the risks associated with anesthesia administration and radiation exposure, but they are considered minimally invasive compared to more invasive surgical methods such as open, laparoscopic, and robotic-assisted laparoscopic surgeries, which are indicated only in patients with anatomic anomalies, such as a UPJ obstruction [109].

### 5.1. Goals of Therapy

The goals of therapy in pediatric patients are identical to those of adults: achieve stone-free status while minimizing complications, risks, and number of procedures. Choice of intervention is informed by stone location and size, anatomic factors, comorbidities, risk factors, stone composition, equipment availability, and provider preference.

### 5.2. Stone Clearance Definition

Arguably the most important clinical outcome to measure after surgery is stone clearance, but there is no universal consensus on its precise definition. In one study, Tasian et al. define stone clearance as “resolution of symptoms with clearance of the offending stone on imaging following ureteroscopy, shock wave lithotripsy, percutaneous nephrolithotomy, or spontaneous stone passage” [18]. However, other studies do not use postoperative imaging to assess stone clearance and instead favor assessment using visual inspection during surgery. Moreover, there is no consensus on the size of residual stone fragments that would be permitted while still being considered “stone free”. This is largely because there is also no consensus on what sizes of stone fragments in pediatric patients are large enough to be clinically significant or small enough to be cleared without adverse clinical outcomes.

### 5.3. Radiation

Because children have rapidly growing tissues and are expected to have longer life expectancies than adults, minimizing the level of radiation exposure is of particular importance in pediatric patients undergoing evaluation or treatment for nephrolithiasis. The American Urological Association therefore recommends “ALARA” methods (radiation exposure that is “as low as reasonably achievable”) of assessment and treatment for pediatric patients but does not provide precise, quantifiable guidelines for radiation exposure [104]. Because of widespread efforts to minimize radiation exposure in children, the use of computerized tomography (CT) to evaluate children with nephrolithiasis has been decreasing since 2007 [110]. However, a majority (approximately 52.2%) of children with nephrolithiasis were still evaluated with CT as of 2015.

Furthermore, surgical methods frequently involve radiation exposure itself, as URS, PCNL, and SWL often utilize fluoroscopy. One study found that pediatric patients were exposed to radiation with a median effective dose of 3 mSv and 16.8 mSv for URS and PCNL, respectively, and patients were exposed to a median of one CT scan and three abdominal X-rays, which corresponded to median effective doses of 14 mSv and 2.1 mSv, respectively [111]. The International Commission on Radiological Protection (ICRP) recommends an annual radiation exposure limit of 20 mSv averaged over 5 years, with no single year having an exposure greater than 50 mSv [112]. Between the exposure levels associated with diagnostic imaging, surgical procedures, and follow-up, patients may approach these exposure limits from a single nephrolithiasis therapeutic plan. The potential dangers of these standard practices have necessitated the recent development of practices that minimize radiation exposure.

Safe and effective practices for PCNL, URS, and SWL have been developed that aim to minimize radiation exposure, and many show promise despite their novelty. Sharifiaghdas et al. found that treating pediatric patients aged 3 to 11 with ultrasonography-guided PCNL using adult instruments yielded a stone-free rate (defined as residual fragment smaller than 4 mm) of 83%, had no major complications, and completely eliminated the risks of radiation associated with other surgical methods used to treat pediatric nephrolithiasis [113]. In 2018, Morrison et al. produced preliminary results demonstrating that ultrasound-guided URS can be used safely in children to effectively manage urolithiasis [114]. Eryildirim et al. found that radiation exposure associated with SWL could be minimized to a range of 0.012–0.015 mSv when performed with an associated kidney–ureter–bladder (KUB) imaging modality [115].

### 5.4. Ancillary Procedures/Anesthesia

Children treated surgically for nephrolithiasis often need multiple anesthetics throughout the course of treatment. In pediatric patients treated with URS, a “pre-stent” is frequently placed to facilitate ureteral access when access cannot be obtained initially [116,117]. This increases the number of anesthetics administered to the patient, as removal of the stent often requires a repeat general anesthetic. Similarly, SWL may require retreatment and therefore repeat anesthetic. This increase in the number of anesthetics administered is of particular concern in children due to the potential neurotoxic effects of anesthesia in children with developing brains and nervous tissues. Little data are available to address these concerns, and the potential dangers necessitate the need for further research investigating the effects of anesthetics on children undergoing surgical treatment for nephrolithiasis.

### 5.5. Surgical Antimicrobial Prophylaxis

The AUA has no specific recommendations for surgical antimicrobial prophylaxis (SAP) in pediatric patients, so prophylaxis protocols follow those set forth for all patients undergoing URS or PCNL or for patients undergoing ESWL who are at increased risk of infection. A urine culture should be obtained preoperatively, and the results of the culture should inform preoperative antibiotic prophylaxis. In a recent RAND/UCLA appropriateness method consensus study, Esposito et al. provided the following recommendations for pediatric patients being surgically treated for nephrolithiasis: “SAP with cefazolin (30 mg/kg i.v.; max 2 g) or trimethoprim/sulfamethoxazole (2 mg/kg of trimethoprim component p.o. in patients >6 weeks of age) only before the procedure (within 30 min before incision) is recommended in children undergoing ESWL if they have a history of previous UTI, large stone burden and anatomical abnormalities and in all children with non-ESWL stone manipulation” [118].

### 5.6. Treatment of Asymptomatic Contralateral Stones

The decision to treat small, asymptomatic contralateral stones discovered incidentally during workup for symptomatic stones has long been a subject of debate. However, in a recent multicenter, randomized, controlled trial, Sorensen et al. found that endoscopically removing small (≤6 mm) ureteral or contralateral kidney stones increased mean time to relapse by 75% and reduced risk of relapse by 82% in adult patients over a mean follow-up period of 4.2 years [119]. Thus, the presence of asymptomatic concurrent contralateral stones may portend the need for earlier treatment. Because this study was limited to adults, more data are needed to determine if treatment of concurrent asymptomatic contralateral stones is warranted in children.

### 5.7. Ureteroscopic Management of Upper Urinary Tract Calculi

The AUA recommends either SWL or URS for pediatric patients who are either unlikely to pass a stone or who have failed a trial of observation or medical expulsion therapy [104]. This recommendation cites stone-free rates of 95% and 78% for small (<10 mm) and large (>10 mm) stones, respectively, treated by URS. In comparison, stone-free rates are 87% and 73% for small and large stones, respectively, treated by SWL. URS is a recommended therapy for pediatric patients with a total renal stone burden less than 20 mm, but beyond this, only PCNL and SWL are recommended. In recent years, URS has gained favor for treatment of ureteral calculi and has overtaken SWL as the preferred modality for treatment of renal stones <20 mm in pediatric patients [120]. In particular, URS is favored in stones located in the distal and middle thirds of the ureter and has been shown to be more efficient in treating distal ureteral stones [121,122].

#### 5.7.1. Key URS Equipment

When performing URS, it is imperative that the endourological suite is familiar to the urologist and assisting staff, and a radiolucent operating table and real-time fluoroscopy (or more recently, real-time ultrasonography) are essential. Central to URS is the ureteroscope, which is available in two forms: semirigid and actively deflectable flexible. The semirigid ureteroscope is effective in treating intramural and distal ureteral stones, and it is particularly useful in assessing distal ureteral blockages encountered during retrograde attempts at obtaining guidewire access or catheterization. The actively deflectable flexible ureteroscope is favored in most other circumstances due to its flexibility, steerability, and ability to traverse most of the upper urinary tract, including the lower renal pole calyces. Semirigid ureteroscopes typically have 2.4 F (French size) to 3.5 F working ports, while actively deflectable flexible ureteroscopes typically have 1.8 F to 3.6 F working ports. A basic endourological suite should also include other accessories essential to URS: a variety of guidewires, open-ended ureteral catheters, a dual lumen catheter, basket devices, a holmium/thulium laser lithotrite, ureteral access catheters, ureteral stents, and ureteral access sheaths.

#### 5.7.2. Key URS Operative Considerations

Many of the techniques and practices of URS performed on children are similar to those of URS performed on adults, but it is important to highlight where these practices differ. All ureteroscopic procedures performed on children should be carried out under general anesthesia, and care should be taken to ensure properly sized equipment, such as stirrups, are used and that all sensitive areas and pressure points are adequately padded to prevent unnecessary nerve injury or skin breakdown, which occur more readily in children [120]. Before beginning surgery, a urine culture should be obtained and verified to be sterile, and intravenous antibiotic prophylaxis should be administered. Patients aged more than 1 year can be placed in the standard lithotomy position, but younger patients should be placed in a modified lithotomy position using gel rolls underneath the knees or in an open leg posture using padded leg boards that extend from the table.

Once the child is positioned correctly and all preoperative protocols have been followed, rigid cystoscopy should be performed to visualize the bladder and ureteral orifices. The ureteral orifice should then be intubated with a guidewire or an open-ended ureteral catheter (either 3 F or 4 F). At some institutions, a retrograde ureteropyelogram is performed with the aid of fluoroscopy to identify relevant renal anatomy and the location of the offending stone. A guidewire is then advanced retrograde through the open-ended catheter until it is coiled in the renal pelvis, as verified by fluoroscopy. This permits passage of the ureteroscope and subsequent access into the ureter. The choice of flexible or semirigid ureteroscope is ultimately left to the discretion of the urologist and is typically based primarily on location of the stone and any pertinent anatomic factors. A holmium: YAG laser is most typically used to facilitate stone fragmentation when basket extraction is not possible, but alternatives include the recently introduced thulium fiber laser [123]. Water should not be used as the irrigant, as some reports have indicated anecdotal experiences of children absorbing too much water, resulting in hyponatremia-induced seizures or intravascular hemolysis [120]. Instead, isotonic irrigation solutions warmed to body temperature should be administered throughout the procedure to prevent hypothermia, hyponatremia, and other metabolic disturbances. The decision to leave a postoperative stent in place is left to the discretion of the urologist and is based primarily on the degree of trauma that is visible at the conclusion of the procedure, the number of passes through the ureter with the ureteroscope, the duration of the procedure, and need for ureteral dilation. The AUA strongly discourages “pre-stenting,” or placing of a stent before a URS procedure to facilitate access during URS, as access to the upper urinary tract is usually possible on the first URS attempt [104].

#### 5.7.3. Limitations and Complications

Overall, URS complication rates in prepubertal children have been found to be similar to, if not better than, URS complication rates in adults [117]. However potential complications include ureteral perforation, ureteral avulsion, urinary tract infection, hematuria, pain, and bleeding. If simple ureteral perforation occurs, the procedure should be halted, and a temporary ureteral stent should be placed. If the much more severe, yet quite rare, ureteral avulsion occurs, it is likely that open repair will need to be performed immediately. Fortunately, significant complications such as this are rarely reported. A systematic review by Ishii et al. found the overall complication rate of URS in children to be 7.1% [124].

### 5.8. Extracorporeal Shock Wave Lithotripsy

Extracorporeal shock wave lithotripsy (ESWL) is a non-invasive procedure that relies on high-energy shock waves generated from a spark plug electrode in a lithotripter device to pulverize renal stones into smaller fragments that can pass through the urinary tract with much greater ease [125]. Since its introduction in 1980, ESWL has become one of the principal treatments for nephrolithiasis as it avoids the need for surgical incisions and the insertion of invasive surgical devices for the extraction of stones [126,127]. The use of ESWL in pediatric nephrolithiasis was initially delayed for a few years, until 1986, due to worries of potential adverse outcomes related to children’s organ development [128,129]. However, now, in comparison to adults, children may be considered better candidates for ESWL because of their shorter, more distensible ureters (which allow for better stone clearance rates), their smaller body habitus, and their resultant shorter skin-to-stone distance [130].

According to the European Association of Urology (2022 EAU Guidelines on Urolithiasis), ESWL should be considered first-line treatment for most cases of pediatric nephrolithiasis, with lower success rates for stones with a diameter greater than 10 mm, impacted calculi of the ureter, calcium oxalate monohydrate or cystine calculi, or calculi in pediatric patients with anatomically and structurally difficult to access urinary tracts [67]. In patients with a total renal stone burden of >20 mm, EWSL and PCNL are both reasonable options, but an internalized ureteral stent or nephrostomy tube should be utilized for ESWL [104].

When stone-free rates (SFRs) for ESWL in children were compared in a 2000 study, calculi with a transverse diameter less than 10 mm had a 92% SFR compared to a 76% SFR for stones with a diameter greater than 10 mm [131]. This finding was supported in a 2015 systematic review and meta-analysis investigating the clinical efficacy and safety of ESWL in pediatric stone disease, as the stone-free rate (SFR) of calculi less than 10 mm in size was significantly greater than stones larger than 10 mm with an overall RR of 1.14 [132]. In this same study, the SFR of calculi in the proximal ureter was significantly greater than calculi in the middle or distal aspects of the ureter [132].

ESWL is usually not considered as first-line to treat cystine stones in children because of the relative hardness of these calculi and their resistance to fragmentation with shock waves as demonstrated by the low 3-month SFR of 37.5% in pre-pubertal patients with cystinuria [133]. However, a recent study reported 83% SFR at 3 months with ESWL for children under the age of 2 presenting with cystine lithiasis [134]. Additionally, pediatric patients with a history of urologic anatomical conditions are not ideal candidates for ESWL as SFR for ESWL was only 12.5% for children who had undergone urinary tract reconstruction surgery or were born with congenital conditions affecting their genitourinary system. This was in comparison to an SFR of 67% amongst children without such conditions in a recent retrospective study [135]. Thus, the size, location, and composition of calculi are important factors to consider when choosing ESWL, along with the ability to localize stones in the patient.

#### 5.8.1. Key ESWL Equipment

As mentioned above, ESWL relies on the use of shock waves generated from an electrode in a lithotripter in order to accomplish the goal of stone fragmentation. ESWL can be accomplished with one of three different shock wave generators: (1) electrohydraulic, (2) electromagnetic, or (3) piezoelectric [125]. Electrohydraulic generators use vaporization bubbles, electromagnetic generators use magnetic fields, and piezoelectric generators use the vibration of crystals as current passes through them in order to produce shock waves.

#### 5.8.2. Key ESWL Operative Considerations

Prior to the ESWL procedure itself, the calculus must be localized with either ultrasound or fluoroscopy, with a preference for ultrasound in order to limit and minimize the amount of radiation placed on the pediatric patient. Once the stone’s location is determined, proper positioning of the patient should be established such that there is no interference of shock wave delivery. Most frequently, the patient is placed in a supine or modified supine position. In order to ensure the effective delivery of shock waves, air bubbles and other impedance should be avoided with use of some media such as ultrasound gel [125].

ESWL is usually conducted under general anesthesia. However, with further innovations and advancements with lithotripters, IV sedation or patient-controlled analgesia can be utilized as an alternative to general anesthesia in older, more cooperative children [136].

The optimal rate for shock wave delivery is not well known for pediatric patients as a recent study comparing ESWL at low and intermediate frequency rates of 60 and 90 shock waves per minute, respectively, demonstrated no statistically significant differences between the two groups [137]. However, the low frequency rate of 60 shock waves per minute may be more advantageous than the intermediate frequency rate of 90 shock waves per minute as it is more efficient with the delivery of fewer shock pulses despite a similar time under anesthesia.

Although stent placement is generally not recommended with ESWL because it has not shown to improve SFR or lower the need for additional procedures, stenting may be useful in preventing or, at least, lowering the risk of steinstrasse formation [138,139,140]. Additionally, as aforementioned, either a stent or nephrostomy tube should be placed if the total renal stone burden is >20 mm [104].

#### 5.8.3. Limitations and Concerns

The most common complication of ESWL is hematuria as the shock waves delivered will cause acute trauma, localizing to the site of the calculus. However, the hematuria will resolve spontaneously within a week after the procedure [141,142]. More severe cases of acute injury due to ESWL include subcapsular hematomas and perirenal bleeding [143,144].

Other post-operative complications can be related to the stone fragments produced by the shock waves in ESWL and the resultant renal colic. This can include the formation of steinstrasse or the regrowth of residual stone fragments retained in the urinary tract. Complications related to infections include bacteriuria and sepsis. Other complications from ESWL arise as a result of damage to structures adjacent to the stone location in the urinary tract. These include bowel perforation, hepatic hematoma, splenic rupture, pneumothorax, urinothorax, abdominal aortic rupture, and acute necrotizing pancreatitis [67,142].

In a recent population-based retrospective study in The Health Improvement Network, ESWL to the kidney was found to be associated with a 40% higher risk of incident hypertension [33]. Since urolithiasis is already associated with a higher risk of hypertension, patients treated with ESWL have a twofold increased risk of hypertension in comparison to those without urolithiasis [33,145]. Although children were not participants in this study, the potential risk of incident hypertension should be considered in the pediatric population given their more vulnerable kidneys and their longer lifespan to develop hypertension.

### 5.9. Percutaneous Nephrolithotomy

PCNL is generally indicated for larger-burden stone disease. Historically, urologists have been hesitant to perform PCNL in children because of concerns for complications such as parenchymal damage, bleeding, radiation, and impaired renal function, among other complications. However, PCNL remains a reasonable option for stones greater than 2 cm in size, complete or partial staghorn calculi, and large cystine stones [146]. Additionally, the European Association of Urology (EAU) guidelines recommend PCNL for >1 cm lower renal pole calculi along with kidney and ureteral stones that have failed SWL and RIRS [147]. Other reasonable indications include challenging lower urinary tract anatomy that prevents retrograde access, calyceal diverticulum, malrotated kidneys, and horseshoe kidneys that are untreatable by SWL and URS [148].

With stone-free clearance rates ranging from 70% to 95%, PCNL remains the preferred treatment for large stone burden in children [149]. However, contraindications include uncorrected systemic bleeding disorders, urinary tract infections, tumors in the planned access tract, and concern for malignant renal cancers [147].

#### 5.9.1. Key PCNL Equipment

Generally, dilation up to 24 Fr is accepted among children. However, smaller instrumentation has been associated with decreased bleeding risk with similar success rates [150]. As such, mini-PCNL (generally 14–20 Fr), ultra mini-PCNL (generally 11–13 Fr), and micro-PCNL (generally <10 Fr) have all been introduced [146].

Dilation can be achieved using either serial coaxial dilators or balloon dilators over a guide wire. A working sheath larger than the nephroscope to allow for proper irrigation should be used.

A variety of energy modalities can be used such as holmium/thulium laser and pneumatic or ultrasonic lithotripters. Finally, a nephrostomy tube may be necessary especially for a second-look PCNL for large stone burden, for adequate drainage, and to facilitate further postoperative imaging [151].

#### 5.9.2. PCNL Planning for Children

PCNL and percutaneous renal access should be performed by experienced operators, as surgeon skill and knowledge are crucial to minimize morbidity. A CT scan should be performed prior to PCNL to determine the optimal calyx to access the stone, if at all possible. Additionally, stone size, location, and adjacent structures should be analyzed.

The assessment of and treatment for urinary tract infections should be performed before PCNL. Generally, a urine culture with antibiotic sensitivities should be ordered 2–3 weeks prior to the procedure. Full courses of antibiotics are indicated for a positive culture. Broad-spectrum intravenous antibiotics (such as cefazolin, trimethoprim/sulfamethoxazole, ampicillin, and gentamicin) should be given peri-operatively [118].

Finally, informed consent must be obtained from the consenting parent or guardian, including reviewing potential complications such as bleeding requiring transfusion, delayed renal hemorrhage requiring angioembolization, sepsis, pneumothorax, hemothorax, urinothorax, incomplete stone clearance, and injury to adjacent organs.

#### 5.9.3. Key PCNL Operative Considerations

PCNL is performed under general anesthesia. Attention should be paid to keeping a warm operating room, using warmed isotonic irrigation, maintaining short operative times, proper draping, and monitoring body temperature to decrease hypothermia risk [152].

The posterior calyx is identified for lower pole access, which is the calyx immediately superior to the most inferior calyx on AP fluoroscopy in >90% of patients [153]. To minimize pneumothorax risk, we obtain upper pole access below the 11th rib and in the medial calyx. If CT guidance is necessary for renal access (i.e., in a child with spinal dysraphism or an aberrantly positioned kidney), Interventional Radiology obtains renal access.

We use an 18 Fr balloon dilator—although metallic coaxial dilation and Amplatz dilation can also be used—and PTFE sheath for renal access [154]. The sheath diameter should be larger than the nephroscope size to allow for proper irrigation.

Children’s kidneys—especially infant kidneys—are more mobile than adult kidneys and commonly pushed away when attempting initial needle access and placing the access sheath. Special attention should be paid to the location of the dilator in the collecting system and to the bony landmarks when advancing the sheath under fluoroscopy. 

#### 5.9.4. Limitations and Concerns

A number of common surgical complications should be noted including bleeding, arteriovenous malformation, postoperative fever and infection, injury to adjacent organs, pneumothorax, hemothorax, urinothorax, and incomplete stone clearance, among others. Other complications include intraoperative ones such as loss of percutaneous tract. Rates of any complication vary from 11 to 36% with Clavien 3 complication rates varying from 1 to 14%. The most common complication is transient fever typically occurring in about 31% of children; however, the source is not always microbial. Transfusion rates can be as high as 24% and were associated with operative time, sheath size, and stone burden. Of note, PCNL has not been shown to be associated with loss of kidney function or scarring [155,156].

**Figure 2:**
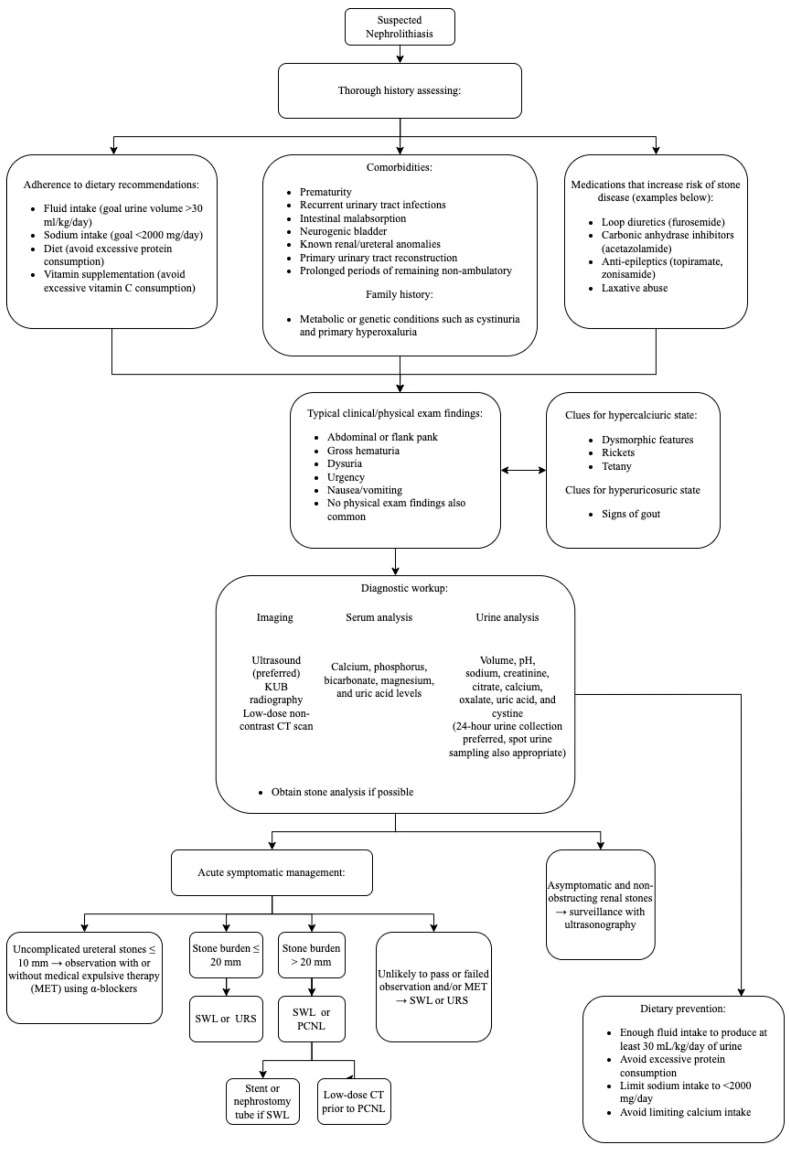
Flowchart summarizing the diagnostic evaluation and management of pediatric nephrolithiasis. Based on AUA guidelines on medical and surgical management of stones [104,158].

If significant intraoperative bleeding occurs, the operation should be stopped, a Foley catheter or re-entry catheter placed through the nephrostomy tract, and clinical treatment started. In the event of renal pelvis injury, the operation should be aborted and, if feasible, an antegrade ureteral stent placed. Treatment of known complications of PCNL in children such as hydrothorax, colonic injury, and postoperative bleeding is similar to adults. Additionally, we recommend that PCNL be performed only at care centers with a pediatric interventional radiology team capable of performing procedures such as bleeding requiring embolization or hydrothorax requiring chest tube [157]. Figure 2 presents a flowchart summarizing the diagnostic evaluation and management (Figure 2).

## 6. Lifestyle Modifications

Finally, various lifestyle modifications can help reduce the risk of developing urolithiasis. Children should drink enough fluid to produce at least 30 mL/kg/day of urine to prevent super saturation of urinary metabolites such as calcium oxalate, calcium phosphate, and uric acid. Studies also suggest that sugary drinks such as soda or fruit juice with high-fructose corn syrup are associated with increased incidence of kidney stones through unclear mechanisms [159]. Many also recommend avoiding excessive protein consumption, as it can increase uric acid formation, hypercalciuria, and hyperoxaluria. Sodium intake should be limited to <2000 mg/day since sodium and calcium compete for passive reabsorption in the nephron. Finally, calcium intake should not be limited, as low-calcium diets can decrease intestinal oxalate absorption, increasing nephrolithiasis risk [158].

Certain behavioral modifications can increase adherence to dietary goals. Medical notes should be provided for more frequent bathroom breaks and for allowing water bottles in classrooms [160]. The Prevention of Urinary Stones with Hydration is a randomized clinical trial assessing the effect of smart water bottle usage on preventing symptomatic stone recurrence, showing the potential for smart technologies to help motivate behavioral changes [161]. Finally, both obesity and lack of exercise have been associated with increased kidney stone incidence in adults; however, this association has not yet been demonstrated in children. Nevertheless, a study by Hannallah et al. stratifying BMI by pubertal status identified possible associations between high BMI and pediatric nephrolithiasis [162].

## 7. Conclusions

In this book chapter, we covered the epidemiology, diagnostic evaluation, medical management, surgical management, and prevention of pediatric stone disease. Key points include the rising incidence, especially in adolescent females; the high burden of disease in a particularly vulnerable demographic; and how a child’s metabolism, growth, and development contribute to the unique pathophysiology of pediatric stone disease. An extensive history, physical, and workup should be performed. Ultrasonography remains the ideal method for imaging followed by either KUB radiography or low-dose non-contrast CT scan. Management consists of observation, medical therapies such as alpha blockers, and surgical intervention—URS, SWL, and PCNL—depending on characteristics such as stone size, pain, comorbidities such as infection, and anatomic considerations. Finally, we reviewed lifestyle modifications that can help potentially prevent stone formation and recurrence.

## Figures and Tables

**Table 1:** Normal 24-h urine lab values of analytes across various age groups *.

Oxalate	<50 mg/1.73 m^2^/day	
Cystine	<60 mg/1.73 m^2^/day	
Citrate	Boys	>125 mg/g creatinine
	Girls	>300 mg/g creatinine
Uric acid	<8 years old	11 mg/kg/day
	8–12 years old	10 mg/kg/day
	>12 years old	7 mg/kg/day
Magnesium	<8 years old	2.5 mg/kg/day
	8–12 years old	1.9 mg/kg/day
	>12 years old	1.8 mg/kg/day
Phosphorus	<8 years old	30 mg/kg/day
	8–12 years old	30 mg/kg/day
	>12 years old	20 mg/kg/day
Creatinine	1–5 years old	20 mg/kg/day
	>5 years old	25 mg/kg/day
Volume	>20 mL/kg/day	

* Lab values vary across institution. Data from Michael et al. [69].

## Data Availability

Not applicable.
